# Milestones in Chronic Lymphocytic Leukemia: An exciting decade of progress—10th anniversary of *memo*

**DOI:** 10.1007/s12254-017-0318-4

**Published:** 2017-02-27

**Authors:** Alexander Egle

**Affiliations:** 3rd Medical Department, Paracelsus Private Medical University Hospital Salzburg, Muellnerhauptstrasse 48, 5020 Salzburg, Austria

**Keywords:** Chronic Lymphocytic Leukemia, Chemoimmunotherapy, Minimal Residual Disease, Ibrutinib, Idelalisib, Venetoclax

## Abstract

The past 10 years have been an exciting ride for Chronic Lymphocytic Leukemia (CLL) aficionados. An overview of changes in management paradigms in CLL, ranging from insights into biology, via chemotherapy and chemoimmunotherapy to maintenance and novel drugs will be presented.

## The path to chemoimmunotherapy

The 10 year anniversary of a journal is not only a reason for celebration, but also provides an opportunity to assess progress in the field over a longer period of time. In our clinical world of incremental improvements, this is a relatively rare opportunity to reflect on what has happened on a larger scale than usual and determine how happy or hopeful that leaves us.

**Fig. 1:**
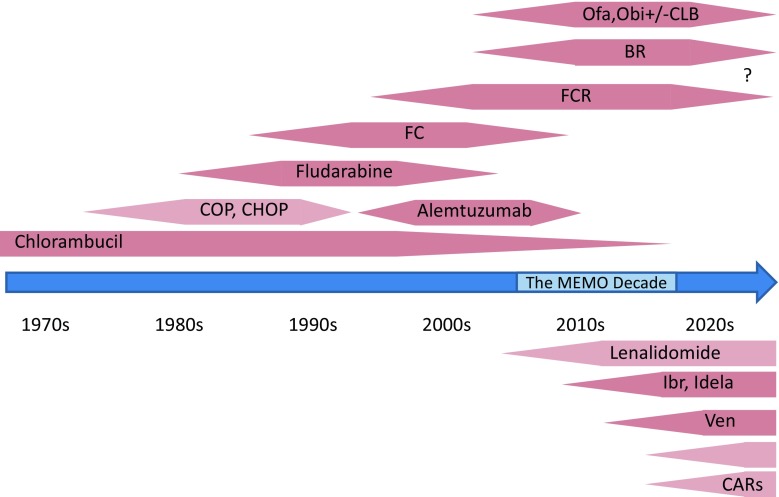
Development of licensed (*dark pink*) and unlicensed (*light pink*) treatment options for CLL over a rough timeline. The *question mark* highlights the possibility that the field may move to chemotherapy-free options. Abbreviations are mainly defined in the main body of the text; *Ofa *ofatumumab, *Obi* obinutuzumab, *Ibr* ibrutinib, *Idela* idelalisib, *Ven* venetoclax, *Combi* novel combinations

About 10 years ago, the CLL field had just left the stage where CLL was treated with chlorambucil (CLB) monotherapy or lymphoma polychemotherapy with limited success for the aggressive variants of CLL for decades. Resurrecting CLL from being the “boring” leukemia, the field developed very relevant dynamics ([1]; Fig. 1). At the turn of the millennium, important biological insights, such as the recognition of recurrent FISH cytogenetic lesions [2], which had a very relevant prognostic impact, or the definition of two biologically distinct groups of CLL, i. e., those with mutated and unmutated IgVH status [3, 4] and again very different clinical behavior, were gained. Also, more effective chemotherapy backbones were proposed and a combination of fludarabine and cyclophosphamide (FC) had been shown to produce better responses and longer PFS in a number of randomized trials [5, 6]. However, none of these trials produced anoverall survival (OS) benefit. In these trials, it also became clear that the proposed FISH cytogenetic groups were able to define subgroups with much worse performance after chemotherapy induction. Thus, del17p and del11q were defined as risk factors with predictive properties towards treatment outcome. At that time, MD Anderson pioneered the use of rituximab in a combination with FC in their typical phase 2 cohort design [7, 8]. Indeed, this combination produced superior outcomes in indirect comparison with historical controls from MD Anderson itself. A little while into the decade, reviewed here, these monocentric data received randomized support from the German CLL 8 trial [9]. This trial not only showed the expected increase in responses and progression-free survival (PFS) for adding the antibody to FC, but was able to define an overall survival benefit for the first time in CLL, thus, changing the standard of care for patients fit to tolerate this intensive regimen. An additional exciting observation from the trial was that patients with del11q (a high-risk group for early failure with fludarabine monotherapy) benefitted substantially from the FCR combination [10].

## The hunt for remission depth

Around the same time, measurement of residual tumor mass—derived either by PCR-based or flow-cytometric methods [11]—showed that patients that had achieved high quality remissions, as defined by these measurements, showed very long remission duration. Initial observations of such minimal residual disease (MRD)-negative states were available from alemtuzumab-treated patients [12]. This very effective antibody was an exciting player at the start of our *memo* decade, but has since fallen by the wayside due to crippling toxicities and management decisions for the drug. However, it remained unclear in phase 2 trials whether the achievement of MRD negativity was merely a sign of “good” risk disease and, thus, associated with superior response or whether it might develop into an independent treatment goal that one could strive to achieve. The concept of MRD as a treatment goal has, indeed, had more staying power than alemtuzumab. In fact, the already mentioned CLL 8 trial had extremely interesting results in this respect [13]: as expected, patients achieving MRD negativity (i. e., <10^−4^ CLL cells) as measured by flow cytometry had significantly longer PFS. This turned out to be independent of the treatment arm, but it became clear that the addition of rituximab was able to roughly double the number of patients achieving MRD negativity, thus, suggesting that remission depth could be an independent treatment goal. Consequently, a number of trials tested whether one could devise more effective treatments, producing higher rates of deep remissions by intensifying therapy. The addition of additional chemotherapy agents (e. g., mitoxantrone) [14] or antibodies (e. g., alemtuzumab) [15] to an FCR backbone was thus tested. The results were, however, relatively disappointing: in the highest risk groups results could not be improved significantly, generating some sort of response plateau. Furthermore, and, equally importantly, the trials demonstrated significantly increased toxicity. With the perspective that FCR was targeted at very fit CLL patients and an option that was already too toxic for a majority of patients with CLL, these escalation trials were, thus, not producing a benefit for a relevant group of patients. Thus, the hunt for MRD negativity took a hit.

## Tailoring treatment intensity and duration

Indeed, a parallel approach to preserve the efficacy of the antibody effects, while reducing the toxicity of chemotherapy backbones, led to the definition of therapeutic options with less intensity than FCR. Combining rituximab with bendamustin (RB) showed encouraging efficacy in phase 2 trials [16], while seeming much less toxic. A randomized comparison (the German CLL 10 trial) [17] was able to confirm this lower toxicity profile of the regimen in fit patients, but showed that the reduction of toxicity came at a price of somewhat reduced efficacy. However, the efficacy benefit of FCR in the trial was not detected in the older patients in the trial. In parallel, the toxicity difference was also very prominent in the older, but fit population. This suggested that for an older or possibly less fit population, RB may have a better risk/benefit ratio than FCR, while maintaining relevant disease control. Parallel approaches in less fit patients showed that the addition of a CD20 antibody (either of rituximab, ofatumumab, or obinutuzumab) [18, 19] was able to improve responses and PFS, and, in the case of obinutuzumab, also a benefit in overall survival. Thus, within a few years after the launch of *memo*, the landscape of treatment in CLL was turned over with the introduction of chemoimmunotherapy regimens that could be tailored to individual tolerability. Since then, a number of approaches to investigate remission maintenance strategies after chemoimmunotherapy induction have been investigated and an improvement of PFS by maintenance with two different CD20 antibodies has been reported in full papers [20, 21]. Similar improvements with lenalidomide maintenance strategies have recently been presented at ASH 2016. None of the trials have been able to report OS benefits for maintenance strategies, but this may be due to short follow-up and/or improved salvage therapies. In fact, we may not be able to fully resolve these questions in the future, given the changes in the field that dominated the last years of the *memo* decade.

## The development of novel treatment paradigms

All the developments described so far had left the highest risk patients—those with del17p and p53 dysfunctions—almost untouched. These patients who had low response rates and short PFS even with intensive induction treatment options, such as FCR. The only patients in this group who were able to achieve longer survival were in the small group of CLL patients who qualify for allogeneic transplant [22]. Some success in this group had been reported for alemtuzumab therapies (e. g., in combination with high-dose corticosteroids and at a high price regarding infections), but that was swept away by things to come.

In parallel to the increase in interest in treating CLL, there was a surge in understanding of the disease biology. Importantly, it became clear that CLL was not merely genetically programmed to proliferate and survive, but relied extensively on microenvironmental interactions for these outcomes [23]. It thus became clear that there would be signaling pathways that provide essential signals for the development of CLL and that these signaling pathways may serve as important targets for the development of treatment. The kinase inhibitors ibrutinib and idelalisib, targeting components involved in the B cell receptor signaling cascade, both showed very interesting phenomenology of response [24, 25]. In fact, both drugs were able to rapidly shrink lymph node masses even in massively pretreated patients (a feat that chemoimmunotherapy has very big problems with). However, apparently the inhibitors did not kill the CLL cells rapidly, but rather spilled them from the lymph nodes (and bone marrow) into the peripheral blood, where a sometimes dramatic increase in lymphocytosis could be observed. This lymphocytosis then decreases over time and the drugs produce a very high rate of partial, but durable remissions under continuing treatment. Also there is a clear tendency for improvement of response qualities over time. Complete remissions, or even MRD negativity, however, remain rare. Thus, the currently used treatment paradigm for these drugs is to treat until progression. The tolerability of the drugs is good compared with chemoimmunotherapy, but both substances have a distinct set of rare side effects that mandate specific management. Excitingly the control of clones with functional p53 deficiency by these drugs is much better than with standard treatment, giving this subgroup important avenues to improved survival [26, 27]. However, with longer observation times the impression is that pretreated and/or p53 deficient CLL has a steady rate of relapse even from these treatments [28]. Thus, alternatives are still needed. Most recently, direct targeting of the cell death machinery, via the Bcl-2 specific “BH3-mimetic” venetoclax has entered the field. Venetoclax produced relevant disease control in pretreated patients and in patients with del17p, adding another option to the armamentarium [29]. Importantly, venetoclax was effective in patients previously treated with kinase inhibitors, giving those patients a salvage option (only presented in abstract form so far). In initial experience, venetoclax led to a high rate of relevant tumor lysis syndromes, but since a slow ramp up of the dose has been mandated this has not proven to be a big problem. Outside of the spectrum of currently licensed options, there are highly relevant developments in the area of immunomodulation (e. g., lenalidomide) [30, 31] and immunotherapy (e. g., CAR T cells) [32] that need to be mentioned but cannot be discussed in the brevity of the overview.

We have, thus, arrived at an exciting moment in CLL history, with lots of options to develop in the future and quite a bit of uncertainty about the optimal treatment pathways of today and tomorrow. As combination approaches with novel drugs are in development, we face the tangible possibility of cure or very long-term control for our patients. This has clearly been a very exciting decade for those interested in CLL, leaving the community both happy and hopeful.
